# The Role of Dicer in DNA Damage Repair

**DOI:** 10.3390/ijms131216769

**Published:** 2012-12-07

**Authors:** Kai-Fu Tang, Hong Ren

**Affiliations:** Key Laboratory of Molecular Biology for Infectious Diseases of the State Ministry of Education, Institute for Viral Hepatitis, The Second Affiliated Hospital of Chongqing Medical University, Chongqing 400010, China

**Keywords:** dicer, DNA damage, tumorigenesis

## Abstract

Dicer is the key component of the RNA interference pathway. Our group and others have reported that knockdown or knockout of Dicer leads to DNA damage in mammalian cells. Two groups recently showed that efficiency of DNA damage repair was greatly reduced in Dicer-deficient cells and that Dicer-dependent small RNAs (~21 nucleotides) produced from the sequences in the vicinity of DNA double-strand break sites were essential for DNA damage repair. Moreover, accumulating data have suggested that miroRNAs play pivotal roles in DNA damage repair. In this review, we discuss the molecular mechanisms by which loss of Dicer leads to DNA damage, as well as the role of Dicer in tumorigenesis.

## 1. Introduction

In 1998, Fire and colleagues reported that double-stranded RNA (dsRNA) was substantially more effective in producing interference than either strand was individually [1]. Specifically, purified single strands were shown to have modest effects after injection into adult animals, whereas double-stranded mixtures caused potent and specific interference [1]. This phenomenon, known as RNA interference (RNAi), is characterized by the presence of small RNAs that are approximately 21 or 22 nucleotides (nt) in length and homologous to genes being suppressed [2–4]. Long dsRNAs are processed by Dicer into 21- and 22-nt short interfering RNAs (siRNAs) that serve as guide sequences to instruct a multicomponent nuclease, the RNA-induced silencing complex or RISC, to destroy homologous messenger RNAs (mRNAs) [3,5].

In addition to siRNA production, Dicer is also essential for the biogenesis of microRNAs (miRNAs). Primary miRNAs (pri-miRNAs), mostly transcribed by RNA polymerase II (RNAPII) [6,7], are processed into a stem-loop structure containing 60- to 70-nt precursor miRNAs (pre-miRNAs) by a microprocessor consisting of an RNAase III endonuclease, Drosha, and a dsRNA-binding protein, DGCR8 [8]. The pre-miRNAs are then transported out of the nucleus via exportin-5 and processed by Dicer into mature miRNAs of 21 or 22 nt in length. These mature miRNAs subsequently bind to the 3′-untranslated region (UTR) of mRNAs and either promote their degradation or inhibit their translation [6–8].

## 2. DNA Damage is Accumulated in Dicer-Deficient Cells

The RNAi machinery plays important roles in the regulation of heterochromatin structure and function [9–11]. Specifically, loss of *Dicer2* has been shown to result in not only decondensation of heterochromatin but also accumulation of extrachromosomal circular repeated DNAs (eccDNA) [12]. Ligase IV, an essential regulator of nonhomologous end joining, perhaps along with other DNA damage repair machinery, have been suggested to participate in the eccDNA formation in *Dicer2*-deficient cells [12]. These findings imply that in addition to increased accessibility of DNA repair and recombination proteins to repeated DNA caused by heterochromatin decondensation, activation of DNA damage response (DDR) may also contribute to the eccDNA formation in *Dicer2-*mutant cells. Moreover, it has been reported that the temporal control of satellite DNA replication in mouse ES cells is sensitive to loss of Dicer [13]. Misregulation of the timing of DNA replication may cause stalled and collapsed replication forks, which can in turn elicit a DNA damage response [14]. Furthermore, the RNAi machinery is known to be essential for transposon silencing [15–19]; therefore, loss of key components in the pathway may activate transposons and consequently lead to DNA damage [20]. Based on these observations, we hypothesized that loss of Dicer can induce DNA damage [21,22]. To test this hypothesis, we knocked down Dicer in human cells. The immunostaining assays for the phosphorylated form of histone H2AX (γ-H2AX; a widely used marker for double-strand DNA breaks [22]) and the replication protein A 70 (RPA70; a protein that becomes phosphorylated and forms intranuclear foci upon exposure of cells to DNA damage [23]) demonstrated that a much higher percentage of Dicer-knockdown cells displayed intense γ-H2AX and RPA70 foci than the control [21]. Using a comet assay to directly assess DNA damage, we confirmed that Dicer knockdown resulted in the accumulation of DNA breaks [21]. Consistent with our results, Peng and Karpen showed that *Drosophila* mutants lacking *Dicer2* displayed significantly elevated frequencies of spontaneous DNA damage in heterochromatin [24]. Mudhasani and colleagues also reported that loss of Dicer activated a DNA damage checkpoint, upregulated the p19(Arf)-p53 signaling, and induced senescence in primary cells [25]. Moreover, Teta and colleagues found that deletion of Drosha or Dicer in rapidly proliferating follicular matrix cells led to DNA damage [26]. Furthermore, 2 groups have reported that knockdown of *Dicer* and *Ago2* resulted in hypersensitivity to UV or gamma irradiation [27,28].

## 3. Molecular Mechanisms by Which Loss of Dicer Leads to DNA Damage

### 3.1. Accumulation of DNA Damage in Dicer-Deficient Cells Is Attributed to Reduced Efficiency of DNA Damage Repair

Wei and colleagues recently reported that the efficiency of DNA damage repair was greatly reduced in *Arabidopsis* with mutations in Dicer-like genes (*dcl2*, *dcl3*, and *dcl4*) and *ago2* alleles and that knockdown of *Dicer* and *Ago2* in human cells inhibited DNA damage repair [29]. In addition, Francia and colleagues reported that in humans, mice, and zebrafish, Dicer and Drosha are necessary for activating DDR upon exogenous DNA damage and oncogene-induced genotoxic stress [30]. The results from RNA deep sequencing further revealed that small RNAs (~21 nt) were produced from the sequences in the vicinity of DNA double-strand break (DSB) sites. These RNAs have been referred to as DSB-induced small RNAs (diRNAs) or Dicer- and Drosha-dependent small RNAs (DDRNAs) [29,30]. Moreover, Francia and colleagues reported that DDR foci formation was sensitive to RNase A treatment and that DDRNAs, either chemically synthesized or generated *in vitro* by Dicer cleavage, were sufficient to restore DDR in RNase-A-treated cells [29,30]. Wei and colleagues also proposed that diRNAs may function as guide molecules to direct either chromatin modifications or the recruitment of protein complexes to DSB sites to facilitate repair [29]. Based on these observations, we suggest that the accumulation of DNA damage in Dicer-deficient cells may be attributed to reduced efficiency of DNA damage repair.

### 3.2. Is Accumulation of DNA Damage in Dicer-Deficient Cells the Consequence of Heterochromatin Decondensation?

It has been demonstrated that chromatin plays pivotal roles in DDR and that disruption of chromatin structures leads to genome instability [31,32]. Specifically, Peng and Karpen reported that *Drosophila* cells lacking the H3K9 methyltransferase Su(var)3-9 showed significantly elevated frequencies of spontaneous DNA damage in heterochromatin and that the accumulation of such DNA damage correlated with chromosomal defects, such as translocations and loss of heterozygosity [33]. In addition, our group demonstrated that inhibition of DNA methylation by 5-aza-2′-deoxycytidine induced DNA damage in human cells [34]. Because loss of Dicer leads to heterochromatin decondensation [10,11], it is interesting to speculate that DDR activation in Dicer-deficient cells is the consequence of heterochromatin decondensation [35]. However, it is worth mentioning that although extensive studies have demonstrated that the RNAi machinery plays a pivotal role in heterochromatin formation in other species, its role in mammalian cells is still controversial [36]. Therefore, whether DDR activation in Dicer-deficient mammalian cells is the consequence of heterochromatin disruption needs to be further investigated.

### 3.3. Is Accumulation of DNA Damage in Dicer-Deficient Cells the Consequence of Transposon Activation?

The RNAi machinery has been postulated to function as the immune system of the genome to defend against molecular parasites, such as transposons and viruses [15–19]. Loss of the key components of the RNAi pathway was shown to activate transposition [15–19], which in turn generated double-strand DNA breaks and elicited DDR [20]. Therefore, we have proposed that activation of transposition may contribute to DNA damage accumulation in Dicer-knockdown cells [21,35]. We reasoned that loss of Dicer stabilizes the transcripts derived from transposons and retrotransposons, thereby causing a high level of transposition and generating double-strand DNA breaks [21,35]. However, whether Dicer can process transposons- and retrotransposons-derived transcripts remains to be elucidated. While it has been reported that endogenous siRNAs derived from SINE/B1 RNAs may exist in mouse cells [37–41] and that decreased Dicer expression led to accumulation of Alu RNAs in human retinal pigmented epithelium cells [42], we found that Dicer may not be able to process Alu RNAs [43]. Therefore, further studies are necessary to address whether Dicer can suppress transposition in mammalian cells.

### 3.4. Is Accumulation of DNA Damage in Dicer-Deficient Cells the Consequence of miRNA Downregulation?

In response to DNA damage caused by ionizing or UV radiation, the expression of cellular miRNAs undergoes global alteration [28,44–46]. DDR can regulate miRNA expression at the transcriptional level; for example, the miR-34a primary transcript has been shown to be directly transactivated by p53 following DNA damage [47–49]. DDR can also regulate miRNA expression by modulating the miRNA processing and maturation steps. Suzuki and colleagues reported that in response to DNA damage, p53 interacted with the Drosha/DGCR8 processing complex via an association with the RNA helicase p68 and facilitated the processing of pri-miRNAs to pre-miRNAs [47,50]. Zhang and colleagues also found that upon DNA damage, the ATM kinase directly binds to and phosphorylates KSRP, thereby leading to not only an enhanced interaction between KSRP and pri-miRNAs but also increased KSRP activity in miRNA processing [51]. Moreover, Pothof and colleagues have proposed a timing model for DDR: DNA damage can induce a fast response (which mainly depends on the regulation of protein activity and/or stability by DNA damage responsive kinases) and a slower response (namely, the transcriptional reprogramming of gene expression). They suggested that DNA damage responsive microRNAs act between the fast protein action and the slower transcription processes [28,52]. These authors also envisaged that the action of microRNAs early in DNA damage response prevents *de novo* protein synthesis, which would otherwise initiate a futile cycle of protein synthesis and degradation or inactivation [52]. Besides regulating cell cycle and cell death, DNA damage responsive microRNAs can also target the key components of the DDR pathways and regulate DNA damage repair [47,52]. For example, miRNA-138 and miRNA-24 have been demonstrated to directly target the 3′-UTR of histone H2AX and repress its expression. Overexpression of miR-138 or miR-24 was also shown to enhance cellular sensitivity to DNA-damaging agents [53]. In addition, miRNA-421 and miRNA-18a can inhibit the ATM expression by targeting the 3′-UTR of ATM transcripts. Ectopic expression of miR-421 or miRNA-18a has also been shown to result in increased sensitivity to ionizing radiation [54,55] as well as reduce the efficiency of homologous recombination-mediated DNA repair [54]. Based on these observations, we propose that loss of miRNAs may affect DNA damage repair [35].

## 4. Dicer, DNA Damage, and Tumorigenesis

Compared to normal tissues, tumor tissues exhibit a general downregulation of miRNAs [56]. Similarly, the Dicer mRNA and protein levels, although still controversial, have been frequently found to be downregulated in tumor tissues [57–59]. The analysis of human cancer genome copy number data has also revealed frequent deletion of Dicer [60]. In addition, Heravi-Moussavi and colleagues recently reported that mutations in the RNase IIIb domain of Dicer are frequently associated with nonepithelial ovarian tumors [61], in which the mutations are restricted to codons encoding metal-binding sites within the RNase IIIb catalytic centers that are critical for microRNA biogenesis [61]. Signs of DDR, including phosphorylation of histone H2AX and Chk2, accumulation of p53, and focal staining of p53-binding protein 1, have been widely observed in clinical specimens from different stages of human tumors and precancerous lesions, but not in normal tissues [62–65]. Since decreased Dicer expression elicits DNA damage [21,24,25], we have raised the following questions: Is there an association between DNA damage and Dicer downregulation in cancer tissues? If the answer is yes, what is the causal relationship between the two in the process of tumorigenesis [35]?

Kumar and colleagues have proposed that loss of Dicer promotes tumorigenesis [66]. They showed that Dicer-knockdown cancer cells had a more pronounced transformed phenotype in animals; that is, Dicer-knockdown cells formed more invasive tumors with accelerated growth than the control tumor cells. In addition, these authors demonstrated that conditional deletion of Dicer enhanced tumor development in a mouse model of K-Ras-induced lung cancer [66]. The same group also suggested that Dicer functions as a haploinsufficient tumor suppressor gene, because full loss of *Dicer1* expression caused inhibition of tumorigenesis [60]. In contrast, Ravi and colleagues recently showed that full loss of *Dicer1* did not preclude tumor formation [67]. They found that transformed or immortalized *Dicer1*-null somatic cells can be isolated readily *in vitro* and that these cells maintained the characteristics of *Dicer1*-expressing controls and remained stably proliferative. In addition, *Dicer1*-null cells from a sarcoma cell line, though depleted of miRNAs, were competent for tumor formation [67]. Similarly, Sekine and colleagues, by disrupting Dicer in hepatocytes using a conditional knockout mouse model, found that Dicer elimination induced hepatocyte proliferation and overwhelming apoptosis [68]. Unexpectedly, they further found that two-thirds of mutant mice spontaneously developed hepatocellular carcinomas (HCCs) at 1 year of age. Deletion of Dicer in thyroid follicular cells has also been shown to cause hypothyroidism with signs of neoplastic alterations [69]. The thyroid tissue in Dicer mutant mice showed marked proliferation of follicular cells as well as an ongoing de-differentiation in the center of the thyroid gland, with loss of Pax8, FoxE1, Nis, and Tpo expression. These observations all suggested that reduced Dicer expression may be a cause of tumorigenesis. Considering the facts that the majority of Dicer-deficient hepatocytes undergo apoptosis and that only a minor subset of them give rise to HCCs, Sekine and colleagues speculated that a “second hit” was required to promote hepatocarcinogenesis in Dicer-deficient hepatocytes [68]. Consistent with this speculation, Kim and colleagues recently reported that Dicer-Pten double-knockout mice universally developed early serous carcinomas in the fallopian tube [70].

Taken together, we have proposed a model to explain the role of Dicer in tumorigenesis [35], in which decreased Dicer expression induces DNA damage and in turn leads to either cell apoptosis or senescence. Alternatively, DNA damage may result in DNA mutations, and cells that contain oncogenic mutations may escape from apoptosis and senescence and eventually form cancers. Furthermore, decreased Dicer expression leads to a global downregulation of cellular miRNAs; since some miRNAs, such as miR-34a and miR-16, may function as tumor suppressors [48,49,71,72], downregulation of such tumor-suppressive miRNAs could also contribute to tumorigenesis.

## 5. Prospects

The role of Dicer in DNA damage repair and cancer etiology has just begun to surface, and the molecular mechanisms by which loss of Dicer leads to DNA damage still need to be further investigated. While the current data implicate that the Dicer- and Drosha-dependent small RNAs (~21 nt) are involved in homologous recombination-mediated DSB repair [29,30], it is important to address the role of these small RNAs in nonhomologous end joining-mediated DSB repair. Moreover, *in silico* predications suggested that several miRNAs could target genes in the DNA damage repair pathways [71]; however, these predications should be further verified experimentally. Another important issue is to address the roles and detailed molecular mechanisms of Dicer and DNA damage responsive microRNAs in cancer development, which will serve as a starting point for developing novel diagnostic and therapeutic strategies for cancer treatment.
